# Advancing resilience in healthcare professionals: A multifaceted IoMT-based strategy to combat burnout

**DOI:** 10.1192/j.eurpsy.2025.1582

**Published:** 2025-08-26

**Authors:** M. A. Lafraxo, H. Guider, A. El Alaiki, Z. Boumaaize, I. Douelfiqar, Y. El Madhi, A. Soulaymani, A. Mokhtari, H. Hami

**Affiliations:** 1 Higher Institute of Nursing Professions and Health Techniques, Oujda; 2 Laboratory of Biology and Health, Faculty of Science, Ibn Tofail University; 3 Laboratory of Electronic Systems, Information Processing Mechanics and Energetics, Faculty of Science, Ibn Tofail University, Kenitra; 4 Regional Center for Education and Training Professions, Rabat, Morocco

## Abstract

**Introduction:**

The escalating burden of occupational stress and chronic health issues among healthcare professionals frequently culminates in burnout syndrome.

**Objectives:**

This study proposes the development of a prototype mobile recommendation system that utilizes the Internet of Medical Things (IoMT) for health monitoring and burnout assessment, while simultaneously offering coping strategies for healthcare professionals.

**Methods:**

A digital framework was conceptualized using the Unified Modeling Language (UML), **which outlined** the system’s modules and graphical interfaces. The proposed system **integrates a range of** biometric sensors, including an **electrocardiogram** (ECG), a blood pressure monitor, a blood oxygen saturation monitor, and temperature sensors, complemented by a psychometric **assessment tool** to evaluate burnout syndrome.

**Results:**

The **results of this study demonstrate that the proposed** system **can offer significant** benefits **in terms of** promoting physical and mental well-being. By **functioning** as a **continuous** health monitoring tool and a preventive measure against stress and chronic illnesses, **the proposed system can enhance** the resilience of healthcare professionals.

**Conclusions:**

The proposed system not only facilitates proactive health surveillance but also delivers targeted interventions to alleviate burnout effects. **The implementation of** this technology could revolutionize mental health management in the workplace, paving the way for robust health policy formulation within medical institutions.

**Disclosure of Interest:**

None Declared

